# А rare case of primary malignant melanoma of the female urethra

**DOI:** 10.1016/j.eucr.2023.102350

**Published:** 2023-02-09

**Authors:** G. Raycheva, A. Ivanov, Zh Chitalov, N. Dimov, V. Popov, P. Antonov

**Affiliations:** a Medical University, Plovdiv, 15, Vasil Aprilov str., 4002, Bulgaria; b Clinic of Medical Oncology, University Hospital “St. George”, Plovdiv, Bulgaria; c Clinic of Urology, University Hospital “St. George”, Plovdiv, Bulgaria; d Clinic of Nephrology, University Hospital “St. George”, Plovdiv, Bulgaria; e Clinic of Radiation Oncology, University Hospital “St. George”, Plovdiv, Bulgaria

**Keywords:** Malignant melanoma, Female urethral cancer

## Abstract

Primary malignant melanoma of the female urethra is an extremely rare disease. Its frequency is only 0.2% of all malignant melanomas. This type of carcinoma is associated with a poor prognosis and short survival due to the early occurrence of metastases and delayed diagnosis. We present a patient with primary malignant melanoma invading the distal urethra, part of the labia minora, and sections of the anterior vaginal wall, where 15 months of overall survival were achieved against the background of the complex treatment.

## 1 Introduction

Primary malignant melanoma of the lower urinary tract is a rare disease with an incidence of less than 1%. Characteristic localizations are the areas of the distal urethra and meatus. In women, it is mainly seen in the sixth to seventh decade of life, but it also occurs in younger patients.^1^^,^^2^ The clinical manifestation is non-specific - bleeding, dysuric disturbances, and tumor formation. This is one of the reasons why the disease is difficult to diagnose, even in an advanced stage.^3^

## 2 Case presentation

**Fig. 1:**
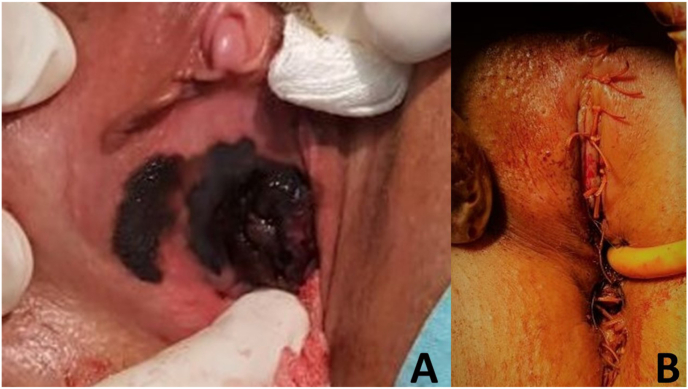
A. Tumor lesion in urethra and part of vagina. B. Result after radical excision.

**Fig. 2:**
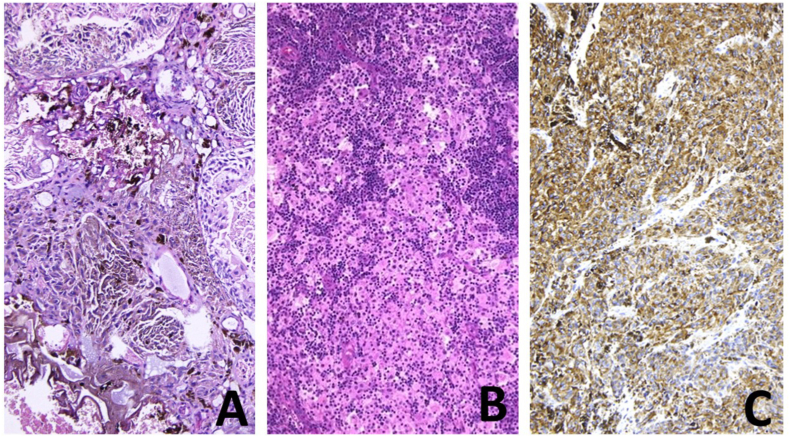
Histology specimen A. Primary tumor (HE) B. Negative for melanoma lymph nodes (HE). C. Infiltrated by melanoma tissue (transurethral resection, HE).

**Fig. 3:**
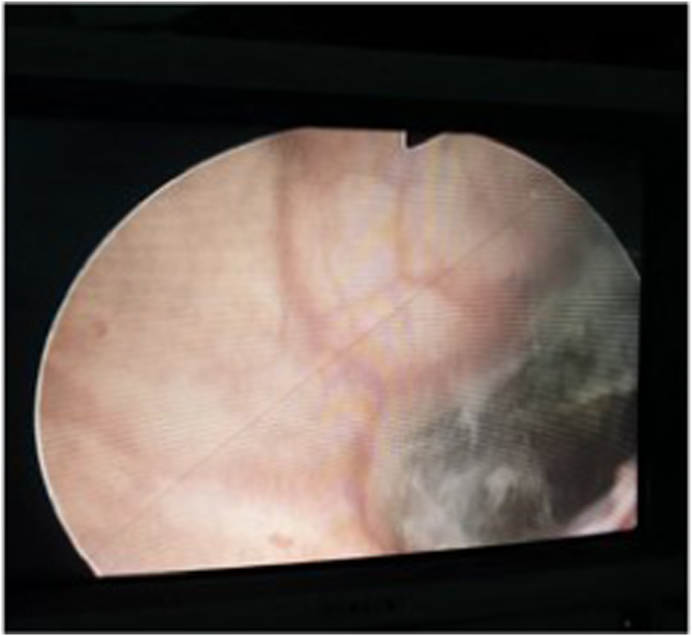
Melanoma in urinary bladder (cystoscopy).

We present a 55-year-old female patient who consulted a urologist in March 2020 due to difficult urination and scanty genital bleeding. Physical examination revealed a tumor about 3 cm in diameter, black in color, bleeding on touch, involving the terminal part of the urethra, the labia minora, and parts of the anterior vaginal wall (Fug 1A). Cystoscopy was done in time of initial diagnosis of disease. The tumor was only in distal 1/3 of the urethra. A biopsy was taken and histologically verified as malignant melanoma. An MRI of the pelvis and a CT scan of the chest, abdomen, and pelvis showed enlarged inguinal lymph nodes bilaterally without distant metastases. In April 2020, surgical treatment was undertaken - vulvectomy, resection of the anterior vaginal wall, partial resection of the urethra, and regional lymph dissection (Fig. 1B). After the procedure voiding was spontaneously and patient was continent. Pathology specimen was with negative tumor margins and inguinal lymphadenectomy was also negative for melanoma invasion (Fig. 2A and B) An oncology committee has evaluated it for radiotherapy and immunotherapy. 60 Gy radiotherapy was administered to the tumor bed, and regional lymph nodes and Interferon alpha therapy were initiated. In control studies in December 2020 progression of the disease is reported - CT data for three tumor formations of about 12–13 mm in size in the bladder. It was confirmed histologically malignant melanoma (Fig. 2C) after transurethral resection of detected masses (Fig. 3). Due to negative BRAF and C-KIT mutational status, platinum-based chemotherapy was started. Four courses of CT were conducted with pronounced hematological and gastrointestinal toxicity. A PET CT was performed in June 2021 with evidence of lung, spleen, and liver metastases. The patient refused further treatment.

## 3 Discussion

Primary malignant melanoma of the urethra is three times more common in women with an average age of onset of the disease around 64. Symptoms include vaginal bleeding, hematuria, dysuria, and tumors on the external genitalia. About 1/5 of the tumors are amelanocytic, which, together with a non-specific clinic, are the leading reasons for late diagnosis.^4^ Primary malignant melanomas of the urethra tend to metastasize early, mainly via the lymphatic route to the vulva and vagina, but also give distant hematogenous metastases to the liver, lung, and spleen. This determines their poor prognosis and short survival.

Urethral malignant melanomas often resemble polyps, which is why they are diagnosed as benign lesions (e.g., caruncles, mucosal prolapse, even lues) or as other malignant neoplasms.

In case of diagnostic difficulties, immunohistochemistry plays a leading role. Melanocyte markers in S-100, SOX-10 and HMB-45 proteins were investigated. A monoclonal antibody specific for S-100 protein is a sensitive marker that reacts with more than 90% of melanomas. Although HMB-45 is specific for melanoma neoplasms, it is less sensitive than the S-100 protein.^5^

Determinants in treating malignant melanoma of the urethra are the size of the primary lesion and the clinical stage. According to the guidelines, in the initial stage of the disease, the method of choice is radical excision with or without radiotherapy. In advanced disease, cystourethrectomy, vaginectomy, and vulvectomy with subsequent dissection of lymph nodes are performed. In the case we presented, we chose a radical surgical approach due to the presence of locally advanced disease. The role of subsequent radiotherapy to the tumor bed and regional lymph nodes has been disputed due to the lack of evidence of an improvement in overall survival in this group, which was also confirmed in our patient.

A leading prognostic factor is the localization of the tumor and its invasion into neighboring organs. Early recurrences and metastases in the liver, lung, and spleen are the reason for the short survival, achieved despite the surgical treatment followed by radiotherapy, immunotherapy, and chemotherapy.

## 4 Conclusion

Primary malignant melanoma is a sporadic disease associated with an aggressive course and short survival. Due to the scarce literature data, a unified approach to therapeutic behavior is lacking. Complex treatment did not significantly improve 5-year overall survival. Histological and immunohistochemical studies could help to make an early and accurate diagnosis of primary malignant melanoma in the urogenital area, improving the long-term prognosis of these patients.

## Declaration of competing interest

The authors declare that they have no competing interest.
